# Longitudinal versus transverse hip arthroscopy portal cosmesis: a case-control trial of simultaneous bilateral cases

**DOI:** 10.1093/jhps/hnz036

**Published:** 2019-09-09

**Authors:** Sina Babazadeh, Matthew J Kraeutler, Tigran Garabekyan, K Linnea Welton, Omer Mei-Dan

**Affiliations:** 1 Australian Orthopaedic Research Group, Melbourne, Australia; 2 Department of Orthopaedic Surgery, St. Joseph's University Medical Center, Paterson, NJ, USA; 3 Southern California Hip Institute, North Hollywood, CA, USA; 4 MultiCare Orthopedics & Sports Medicine, Auburn, WA, USA; 5 Department of Orthopedics, University of Colorado School of Medicine, Aurora, CO, USA

## Abstract

The direction and nature of incisions can impact the healing and appearance of a surgical scar. This can be attributed mainly due to skin tension and direction of force. The aim of this study was to identify differences in healing rates and scar esthetics between transverse and longitudinal portals used for hip arthroscopy. A total of 75 patients underwent bilateral hip arthroscopy for femoroacetabular impingement. All patients received a portal perpendicular to the long axis of the body on the left side (transverse portal) and parallel with the long axis of the body on the right side (longitudinal portal) for the standard anterolateral viewing portal. Postoperatively, patients were reviewed at 2 weeks, 6 weeks, 3 months and 6 months and the portal scars were assessed, photographed and measured. No patients were lost to follow-up. The transverse scars, although slightly longer, were found to be narrower at 6 weeks (3.8 mm versus 2.7 mm, *P* < 0.01), 3 months (4.3 mm versus 3.4 mm, *P* = 0.01) and 6 months postoperatively (6.1 mm versus 4.5 mm, *P* < 0.01). At 3 months (43 mm^2^ versus 35 mm^2^, *P* = 0.029) and 6 months (49 mm^2^ versus 43 mm^2^, *P* = 0.024), transverse incisions were noted to have significantly reduced total area compared with longitudinal incisions. There were no wound complications in either group. This study demonstrates that transverse portal positions for hip arthroscopy have an advantage over longitudinal portal positions in terms of total scar area and thickness up to 6 months postoperatively.

## INTRODUCTION

Hip arthroscopy is commonly used to address intra-articular cartilage and labral pathologies. Entry portal incisions are necessary for the advancement of arthroscopic surgical instruments. In our hands, these incisions are usually 7–15 mm in length with most surgeons using 2–4 portals in most therapeutic procedures [1]. The ultimate goal of any surgical incision is to achieve quick healing while maintaining esthetics, with minimal to no discomfort or long-term associated irritation. Due to inelasticity, incisions for elective orthopedic procedures may result in wide and hypertrophied scars or impeded function [2]. The direction and nature of incisions can impact the healing and appearance of the scar. This can be attributed mainly due to skin tension and direction of force [3]. Arrangement of elastic fibers in the dermis creates skin tension lines and, if incisions are not made along these lines, the resulting scar demonstrates signs of hypertrophy or stretching due to this increased tension [4]. These lines are known as Langer lines [5].

There are many contributors to wound healing including Langer lines, Borges’ theory on relaxed skin tension lines produced by pinching the skin or Kraissl’s preference for lines oriented perpendicular to the action of the underlying muscle [3]. However, when any of these guides to elective incisions are combined with fundamental biomechanical skin properties such as elasticity, strain, creep and stress relaxation, appropriate wound healing ensues.

Clinical studies suggest that the postoperative scar formation is influenced by local wound factors. Through the stages of inflammation, proliferation and remodeling, influenced by mechanical forces including tension and depth, epidermal closure seeks to restore skin integrity [6]. Modifiable factors of incisional scar response include incision design, atraumatic handling of soft tissue, aseptic techniques and tension-reducing approaches in both the short- and long-term postoperative setting [7]. Non-modifiable factors include patient age, ethnicity and previous history of wound healing.

Many techniques have been utilized to improve scar appearance and outcomes with varying results. In laparoscopic and orthopedic surgery, some of these techniques include changing scar length [8], changing suture material and layering [9, 10], altering incision orientation [11] and a move toward minimally invasive surgery [12].

However, evidence-based guidance regarding the optimal orientation for hip arthroscopy portals is lacking. In our experience, due to their short length, arthroscopy portal orientation can be altered relatively easily without affecting surgical outcome. This is in contrast to larger incisions required for open surgery, such as joint replacement, which are often dictated by the underlying anatomy and are more difficult to alter.

The purpose of this study was to identify differences in scar cosmesis and healing complications between transverse and longitudinal portals used for hip arthroscopy. The authors hypothesized that compared to longitudinal incisions, transverse portal incisions heal with improved cosmesis, with a smaller scar and fewer complications, as the portal is aligned parallel with the skin creases and is more closely associated to Langer's lines, reducing the tension vector.

## MATERIALS AND METHODS

This study was done in agreement with the ethical standards of the institutional and/or national research committee and with the 1964 Helsinki declaration and its later amendments. After Institutional Review Board approval was obtained (12-1385), the authors prospectively enrolled a cohort of patients presenting with hip pain to a dedicated hip preservation clinic. Common indications for referral included femoroacetabular impingement, hip instability, acetabular dysplasia and associated abnormalities of femoral torsion or acetabular version. Inclusion criteria for patients selected for this study were as follows: (i) persistent bilateral hip pain and mechanical symptoms refractory to nonoperative management (physical therapy, non-steroidal anti-inflammatory drugs, activity modifications and corticosteroid injections) lasting at least 3 months, (ii) reproducible clinical examination findings suggestive of impingement, (iii) joint-space width exceeding 3 mm on all views of plain radiography and cross-sectional imaging and (iv) no previous hip joint surgery. All patients included in this study had similar pre-arthritic hip conditions bilaterally (cam/pincer/mixed-type femoroacetabular impingement requiring osteoplasty, labral tears, etc.). If a patient was found to have a chondral lesion requiring microfracture during the first hip arthroscopy, arthroscopy of the contralateral hip was delayed until after a 6-week period of non-weight bearing restrictions on the affected hip. Thus, these patients were excluded from this study.

Some of the physical examination tests used included passive hip range of motion (supine, lateral and prone), the FADIR (flexion, adduction and internal rotation) test, the FABER (flexion, abduction and external rotation) test, the ligamentum teres (LT) test, the posterior impingement test, use of the Beighton Hypermobility score and subjective reports of hip instability [13]. Patients who had undergone previous hip surgery, had skin disease or tattoos over the incision area were excluded. Demographic variables including age, clinical diagnosis and gender were recorded for all patients.

### Patient cohort and intervention

**Fig. 1.**
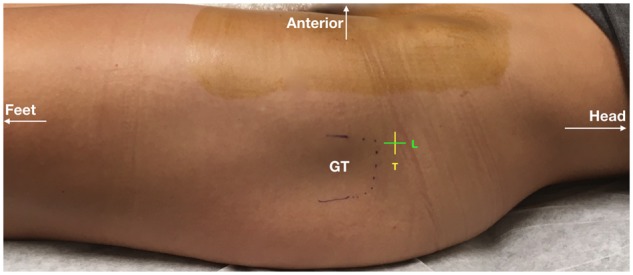
Clinical photograph demonstrating anterolateral portal location on the left thigh and patient orientation preoperatively. In this study, all left hips received a transverse portal and all right hips received a longitudinal portal. L, longitudinal portal; T, transverse portal.

**Fig. 2.**
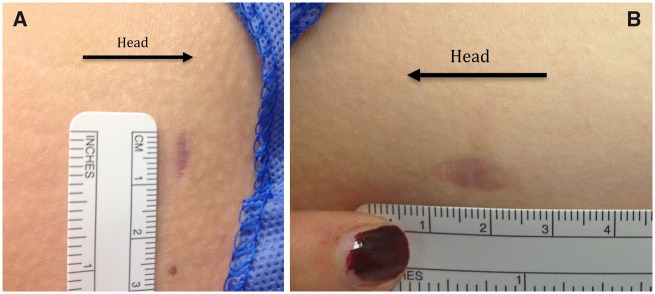
Clinical photographs of incision-site healing at 3 months postoperatively. (**A**) Transverse portal on the left thigh noted to heal with thinner scar compared to longitudinal portal on the right thigh (**B**).

**Fig. 3.**
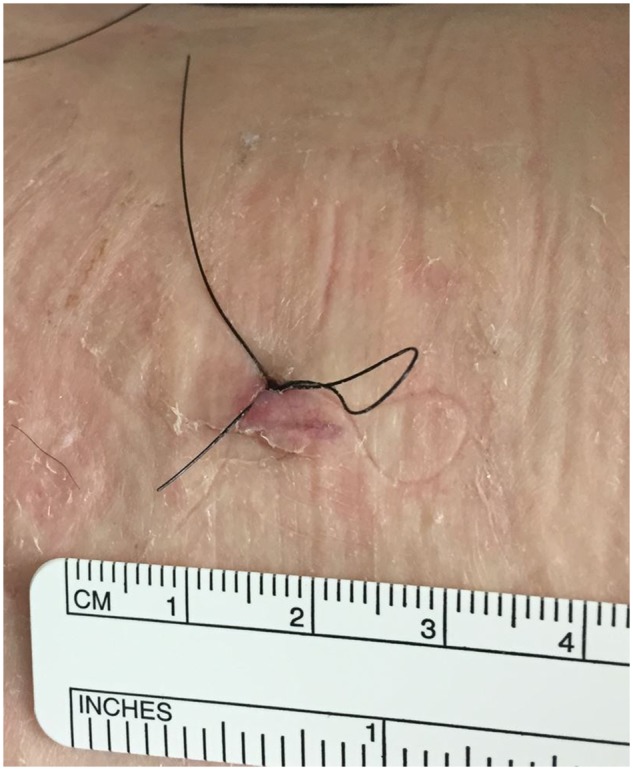
Loop suture closure used to close portals [11].

All patients included in this study underwent simultaneous bilateral hip arthroscopy performed under the same anesthetic, as described previously [14]. Hip arthroscopy was performed without a perineal post [15, 16]. At least two portals were used for each side. Portal locations are demonstrated in Fig. 1. All patients received a portal perpendicular to the long axis of the body on the left side (transverse portal—Fig. 2a) and parallel with the long axis of the body on the right side (longitudinal portal—Fig. 2b). All portals were closed using a looped suture technique using a three nylon non-absorbable suture removed at 10–14 days post-surgery (Fig. 3) [17].

Only the standard anterolateral portal was assessed for this study as this was the standard portal present in all patients. Being the viewing portal, this portal’s length did not change with differing needs for instrumentation with the average length being 12.5 mm (range 8–15 mm).

### Data collection

Demographic data including age, sex and medical history were recorded preoperatively. The skin was also assessed and deemed to be appropriate for inclusion in the study.

Postoperatively, patients were reviewed at 2 weeks, 6 weeks, 3 months and 6 months by the operating surgeon. During these visits, the portal scars were assessed, photographed and then measured by the lead investigator. Length and width were recorded and total surface area was estimated by multiplying the measured length and width. All complications were recorded at these time points.

### Measurements

All portals were photographed at each follow-up visit using a ruler adjacent to each portal for scaling purposes (see Fig. 2). These photographs were then scaled and the portals measured using a computer graphics program (ImageJ, National Institutes of Health) by outlining the scar and laying this next to the ruler to achieve an accurate measure of the portal length and width.

### Statistical analysis

After variables were evaluated for distribution of normality using the Shapiro-Wilk tests, quantitative analysis was performed using an analysis of variance test with repeated measures for mixed data sets. Descriptive statistics were summarized as the mean and standard deviation (SD) for quantitative variables and as counts and frequencies for categorical variables. A *P* < 0.05 was deemed significant for all comparisons. All analyses were conducted using SigmaPlot Statistics software (Systat Software Inc.).

## RESULTS

A total of 75 patients met inclusion criteria and were enrolled into the study. No patients were excluded. No patient was lost to follow-up. The majority of patients were female (73%) with a mean age of 28.5 ± 10.2 years.

No wound complications were noted in any patient. All patients’ scars were completely healed at 6 weeks but continued to mature through 6 months. Steristrips or other adhesive devices were not required after suture removal. No patient required a re-operation due to wound complications.

Mean length and thickness of the portals can be found in Table I. At the 2-week follow-up appointment, there was no significant difference between groups with regard to scar length, width or total area (*P* > 0.830). Transverse scars, although slightly longer, tended to heal with a thinner scar. This trend became significant at 6 weeks (*P* = 0.005) and remained significant at 3 (*P* = 0.010) and 6 months (*P* = 0.002) with scar width being significantly smaller in transverse scars compared with longitudinal scars. At 6 months, scar width was on average 1.35-times wider in the longitudinal group than the transverse group.

**Table I.** Scar length and width measurements

Time-point	Right (longitudinal) Mean (mm) (SD)		Left (transverse) Mean (mm) (SD)		*P* value
	Length	Width	Length	Width	Length/Width
**2 weeks**	9.9 (1.5)	2.8 (1.5)	10.1 (2.1)	2.9 (1.6)	0.885/0.830
**6 weeks**	9.3 (1.6)	3.8 (1.2)	11.3 (1.7)	2.7 (1.0)	<0.001*/0.005*
**3 months**	10.1 (1.7)	4.3 (1.7)	10.8 (2.2)	3.4 (1.3)	0.147/0.010*
**6 months**	10.5 (1.9)	6.1 (1.6)	12.0 (2.2)	4.5 (2.1)	0.008*/0.002*

* *P* < 0.05 was considered statistically significant.

**Fig. 4.**
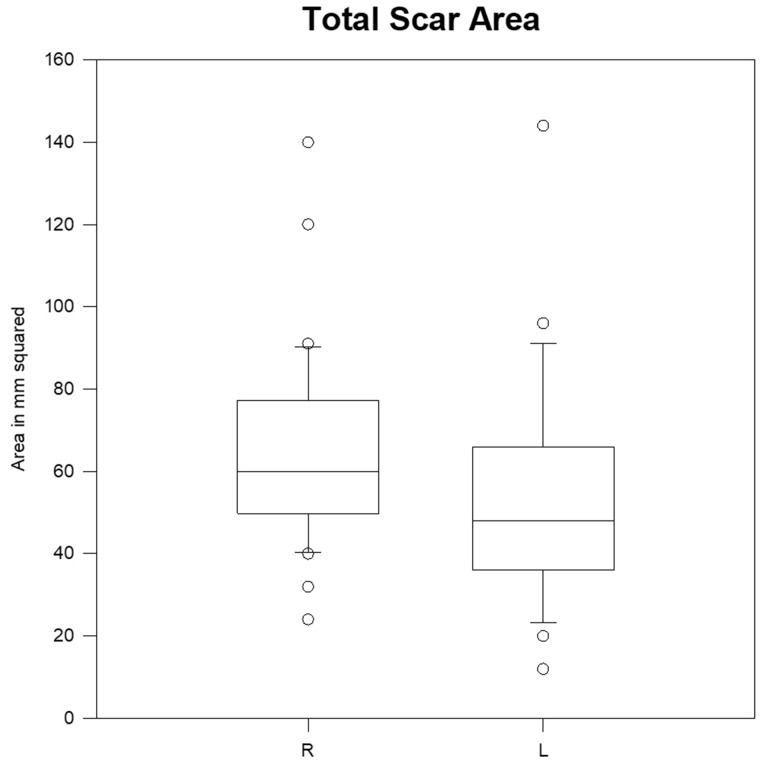
Total area at 6 months in mm^2^. R, longitudinal; L, transverse.

A comparison of total scar area can be found in Table II. At 3 and 6 months, transverse scars were noted to have significantly reduced total area compared with longitudinal scars (*P* = 0.029 and 0.024, respectively) (Fig. 4).

**Table II.** Measurements of total surface area

Time-point	Right (longitudinal) Mean (mm^2^) (SD)	Left (transverse) Mean (mm^2^) (SD)	*P* value
**2 weeks**	27.89 (16.4)	28.78 (14.5)	0.864
**6 weeks**	31.21 (12.3)	30.23 (11.4)	0.703
**3 months**	43.14 (17.9)	35.05 (12.8)	0.029*
**6 months**	48.5 (20.7)	43.35 (18.7)	0.024*

* *P* < 0.05 was considered statistically significant.

When assessing the relationship between scar length and width, no significant correlation was found (longitudinal *P* > 0.619 and transverse *P* > 0.606).

## DISCUSSION

This study demonstrates that transverse portal incisions had a significant advantage over longitudinal portal incisions in terms of total scar area and scar width, resulting in overall improved esthetics. This is the first study assessing differences in hip arthroscopy portal orientation in terms of postoperative healing characteristics.

The esthetic appearance of a scar was typically associated with its width, as visually a thin scar, regardless of its length, was difficult to see and from our general experience, well tolerated among patients. This study also demonstrated that as the scar matured over time, the difference between a longitudinal and transverse scar became more apparent. This deviation may be associated with skin tension lines around the scar, with transverse scars more closely orientated to these natural lines of tension and resulting in a more esthetic scar formation.

The findings of this study are pertinent to our hip arthroscopy population. These patients are often young, athletic and can be self-conscious regarding surgical scars. Anecdotally, many patients commented regarding the difference between scars and their preference for a thinner scar.

Scar size and formation may be improved by aligning them to lines of relaxed skin tension. Transverse portals may be more closely aligned to Langer’s lines and subsequently may be subject to reduced tension. Hence, this may be a reason why they are esthetically superior to longitudinal portals. Furthermore, the movement of instruments in hip arthroscopy is mostly in the transverse direction, hence there may be more trauma incurred to the portal edges when the portals are orientated in the longitudinal direction, providing further reasoning as to why transverse portals heal with a smaller scar.

One major strength of this study is that by only including patients undergoing bilateral, simultaneous hip arthroscopy, the authors were able to control for all known modifiable and non-modifiable risk factors in our study cohort. This neutralizes many of the patient-related factors that can affect wound healing. The limitations of this study should also be noted. In particular, the surgeon could not be blinded to the incision orientation. In addition, although an attempt was made to minimize other patient factors, there may have been slight differences in duration and type of hip pathology between the two sides. It is possible that a surgeon’s hand dominance could have an effect on outcomes of right versus left hip arthroscopy [18]. Measuring only the anterolateral portal also meant that complications such as injury to the lateral femoral cutaneous nerve of the thigh were not pertinent to this study. This is an important complication that often affects only the anterior portal, and hence this study is unable to determine if changing portal orientation at the anterior location may affect this. Finally, patient satisfaction with regard to scar appearance was not assessed in this study.

This study demonstrates that transverse portals have an advantage over longitudinal portals in terms of total scar area and thickness up to 6 months postoperatively.

## FUNDING

No funding was received for this project.

## CONFLICT OF INTEREST STATEMENT

None declared.
